# Loxoprofen overdose without toxicity: pharmacokinetics of loxoprofen and metabolites analysed by liquid chromatography–mass spectrometry: a case report

**DOI:** 10.1186/s12245-026-01256-4

**Published:** 2026-05-29

**Authors:** Daiki Mori, Jotaro Tachino, Kazuo Harada, Miyu Ueki, Kensuke Minami, Ryosuke Takegawa, Tomoya Hirose, Shota Nakao, Hiroshi Matsumoto, Jun Oda

**Affiliations:** 1 Department of Traumatology and Acute Critical Medicine, Graduate School of Medicine, The University of Osaka, 2-15, Yamada-Oka, Suita City, Osaka Japan; 2 Rinku General Medical Center, Senshu Trauma and Critical Care Center, Rinku Orai-Kita 2-23, Izumisano City, Osaka 598-8577 Japan; 3 School of Pharmaceutical Sciences, The University of Osaka, Yamada-Oka 1-6, Suita City, Osaka Japan; 4 Department of Legal Medicine, Graduate School of Medicine, The University of Osaka, Yamada-Oka 2-2, Suita City, Osaka Japan; 5 The Education and Research Institute for Death Control and Prevention, Graduate School of Medicine, The University of Osaka, Yamada-Oka 2-2, Suita City, Osaka Japan

**Keywords:** Loxoprofen, Trans-OH metabolite, cis-OH metabolite, LC/MS, Overdose

## Abstract

**Background:**

Loxoprofen, widely used as a nonsteroidal anti-inflammatory drug worldwide, is a prodrug that is converted to its active trans-hydroxylated metabolite. Reports of loxoprofen overdose are extremely limited, and asymptomatic cases despite massive ingestion have not been previously documented.

**Case presentation:**

A 43-year-old woman ingested 8,280 mg of loxoprofen. She arrived at the hospital two hours later in a clinically stable condition. Laboratory tests and electrocardiography were unremarkable, and abdominal computed tomography revealed no gastric contents. She remained asymptomatic, with normal laboratory findings throughout the hospitalisation period, and was discharged on day 3. Pharmacokinetic analysis using liquid chromatography–mass spectrometry revealed markedly elevated plasma concentrations of loxoprofen and its metabolites two hours after ingestion (loxoprofen 159 µg/mL, trans-OH 64 µg/mL, cis-OH 29 µg/mL), which declined rapidly thereafter.

**Conclusion:**

This case suggests that markedly elevated plasma concentrations of loxoprofen and its metabolites do not necessarily result in toxicity. Pharmacokinetic findings indicate that rapid metabolism may prevent organ damage.

## Background

Loxoprofen is a prodrug converted in the liver by carbonyl reductase enzymes into trans- and cis-hydroxylated metabolites [1]. The active metabolite, trans-OH, exerts anti-inflammatory effects by inhibiting cyclooxygenase and suppressing prostaglandin synthesis [1, 2]. Although loxoprofen is known to cause adverse effects such as renal and hepatic dysfunction and gastrointestinal disturbances [1], reports of overdose are extremely limited. Although a previous report described a patient who was asymptomatic at presentation but later developed toxicity [3], there is no published no report of a patient remaining asymptomatic without organ dysfunction despite elevated plasma concentrations of loxoprofen.

Herein, we report a case of massive loxoprofen ingestion in which high plasma concentrations of loxoprofen and its metabolites were confirmed through liquid chromatography–mass spectrometry (LC/MS), but no toxic symptoms or organ dysfunction were observed.

## Case presentation

### Clinical course

A 43-year-old Japanese woman (72 kg, 151 cm) intentionally ingested 8,280 mg of loxoprofen (138 tablets of Loxonin^®^ 60 mg). Her family noticed that she had ingested an excessive amount of loxoprofen and called emergency medical services: she was transported to our hospital approximately two hours later. The patient had not consumed alcohol at the time of loxoprofen ingestion. Her medical history included bipolar disorder, and she had used loxoprofen intermittently prior to this overdose, although the exact duration of use was unknown.

Upon arrival, her vital signs were as follows: respiratory rate, 20/min; oxygen saturation, 97% on room air; heart rate, 82/min; blood pressure, 143/108 mmHg; and Glasgow Coma Scale score, 15 (E4V5M6). She was alert with no symptoms. Laboratory test results and electrocardiography findings were unremarkable. Computed tomography revealed no gastric contents; therefore, gastric lavage was not performed. Activated charcoal was administered, intravenous fluids were initiated, and she was admitted. Laboratory tests showed no organ dysfunction 8 h post-ingestion, and physical examination revealed no gastrointestinal symptoms or oliguria. Oral intake resumed 13 h after ingestion, and laboratory tests remained unremarkable throughout hospitalisation (Table 1). The patient remained clinically stable and asymptomatic throughout the course. Intravenous fluids were discontinued on day 3, and she was discharged home.

**Table 1:** Laboratory data during hospitalisation

Time after loxoprofen overdose(hour)		2	8	32	56
Blood cell count					
White blood cell	(103/μL)	10.22	7.69	9.21	8.71
Red blood cell	(106/μL)	5.33	4.95	4.72	4.69
Hemoglobin	(g/dL)	14.5	13.2	12.6	12.8
Hematocrit	(%)	44.4	42.1	40.4	40
Platelet	(103/μL)	208	212	186	194
Biochemistry					
Sodium	(meq/L)	138	139	138	137
Potassium	(meq/L)	3.9	4.3	3.9	4.1
Chloride	(meq/L)	103	102	103	103
Calcium	(mg/dL)	8.6	8.2	8.2	8.5
Blood urea nitrogen	(mg/dL)	11	9	9	8
Creatinine	(mg/dL)	0.44	0.45	0.51	0.5
Aspartate minotransferase	(U/L)	28	23	26	22
Alanine aminotransferase	(U/L)	29	25	24	21
γ-glutamyltransferase	(U/L)	25	21	18	18
Alkaline phosphatase	(U/L)	70	61	57	58
Lactate dehydrogenase	(U/L)	215	172	151	130
Total Bilirubin	(mg/dL)	0.2	0.4	0.9	0.8
Creatine kinase	(U/L)	57	48	41	34
Amylase	(U/L)	50	46	40	45
Total protein	(g/dL)	7.2	6.1	5.8	5.9
Albumin	(g/dL)	3.7	3.4	3.2	3.2
C-reactive protein	(mg/dL)	0.41	0.35	0.61	1.61

### Pharmacokinetic analysis

The plasma concentrations of loxoprofen and its metabolites, trans-OH and cis-OH, were determined in collaboration with the Department of Legal Medicine, Graduate School of Medicine, the University of Osaka.

**Fig. 1:**
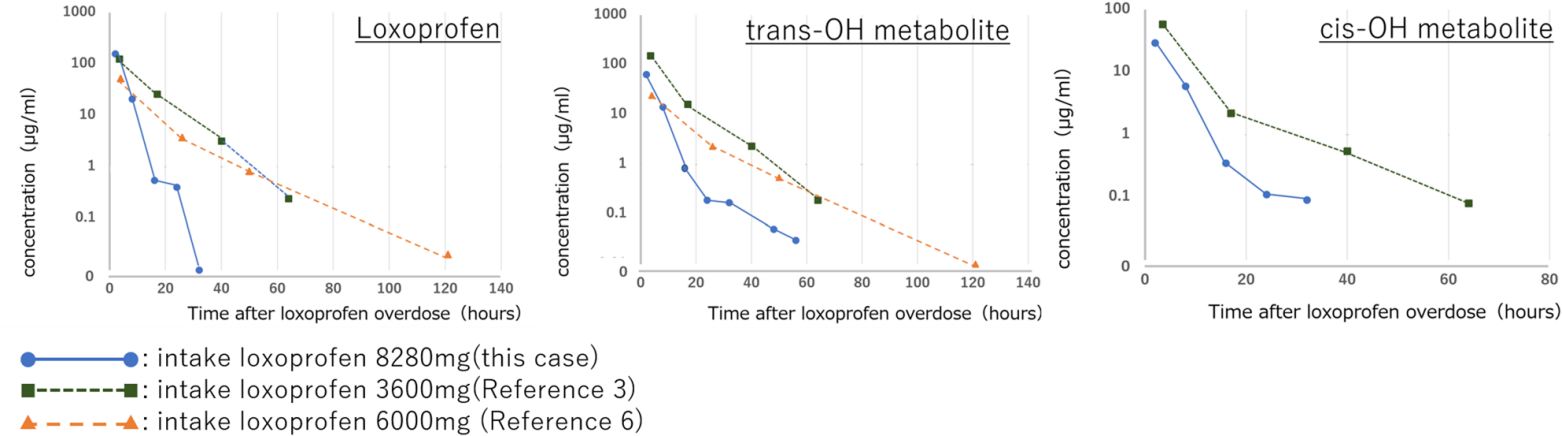
Plasma concentrations of loxoprofen, trans-OH metabolite, and cis-OH metabolite in the present case were compared with those reported in two previous cases. The present case is indicated by circles (○), and previously reported cases are represented by triangles (△) and squares (□)

Blood samples were centrifuged at 1500 rpm for 5 min at 5 ℃ to obtain plasma. Sodium citrate was used as an anticoagulant. Plasma samples were stored at -20 ℃ until pretreatment. Pretreatment of plasma samples (50 µL each) was performed using the dispersive solid-phase extraction QuEChERS method, followed by LC/MS analysis in the multiple reaction monitoring mode. The initial screening of 260 compounds detected the presence of loxoprofen, trazodone, and caffeine [4]. Quantitative analyses were subsequently performed for these compounds, as well as for the trans- and cis-hydroxylated metabolites of loxoprofen, under chromatographic conditions that allowed the separation of the two isomers. Stable isotope-labelled internal standards were used for each target compound: loxoprofen-d₃, trazodone-d₆, caffeine-^13^C₃, trans-OH-d₃, and cis-OH-d₃. The concentrations of loxoprofen, trans-OH, and cis-OH are shown in Fig. 1; Table 2. Trazodone was below the therapeutic range and caffeine was far below the toxic level, indicating no significant co-ingestion.

**Table 2:** Plasma concentrations of loxoprofen, trans-OH metabolite, and cis-OH metabolite after loxoprofen overdose

Time after loxoprofen overdose(hour)		2	8	16	24	32	48	56
loxoprofen	(μg/mL)	159	21	0.55	0.40	0.01	0	0
trans-OH metabolite	(μg/mL)	64	14	0.79	0.19	0.17	0.05	0.03
cis-OH metabolite	(μg/mL)	29	5.9	0.35	0.11	0.09	0	0

## Discussion

We report a case of massive loxoprofen ingestion without clinical toxicity, suggesting that markedly elevated plasma concentrations of loxoprofen and its metabolites may not cause toxicity.

We hypothesised that the absence of toxic symptoms may have been related to the rapid clearance of loxoprofen and its metabolites. After oral administration of 60 mg, the reported maximum plasma concentrations of loxoprofen, trans-OH, and cis-OH have been reported to be 5.04 ± 0.27 µg/mL, 0.85 ± 0.02 µg/mL, and 0.31 ± 0.04 µg/mL, respectively, with corresponding times to reach maximum concentration of 0.45 ± 0.03 h, 0.79 ± 0.02 h, and 0.73 ± 0.06 h [5]. In contrast, in previous cases of 6000 mg and 3600 mg, plasma concentrations reached 52/24 µg/mL(cis-OH not measured) 4 h after ingestion and 126/152/58.1 µg/mL 3.5 h after ingestion for loxoprofen, trans-OH, and cis-OH, respectively, with clinical toxicity [3, 6]. In this case, plasma concentrations measured 2 h after ingestion were 159, 64, and 29 µg/mL, respectively. These levels were comparable to or exceeded those reported in previous studies; nevertheless, no apparent toxic symptoms were observed. The estimated plasma half-lives of loxoprofen (1.7–2.5 h) and trans-OH (2.7–4.9 h) —calculated based on the terminal elimination phase of the observed time points—were shorter than the previously reported range of 6–12 h [3, 6], implying rapid metabolism and elimination before the development of organ dysfunction.

Loxoprofen is mainly metabolised in the liver to trans-OH and cis-OH [1, 5], which are subsequently excreted in urine [5, 6]. Nonsteroidal anti-inflammatory drugs have been reported to cause hepatic dysfunction [7] and inhibit prostaglandin biosynthesis, and may also contribute to renal impairment, particularly under conditions of reduced renal perfusion [8]. Previous reports of loxoprofen overdose suggest that hepatic or renal dysfunction may lead to impaired metabolism and delayed elimination, resulting in reduced drug clearance [3, 6]. In this case, preserved organ reserve in the absence of underlying disease, together with potential genetic factors and the benefit of early supportive therapy, might have contributed to more efficient drug metabolism and excretion. It is noteworthy that, although the concentration of the active metabolite trans-OH—which inhibits prostaglandin biosynthesis and can induce renal ischemia—reached levels comparable to toxic cases, renal function remained preserved. An increase in creatinine clearance is reported to be associated with enhanced loxoprofen clearance from the circulation [2]. We speculate that early initiation of intravenous fluid therapy helped maintain adequate renal blood flow and preserved creatinine clearance in this case, which may have mitigated the risk of hemodynamically mediated acute kidney injury despite the high trans-OH exposure. However, as urinary concentrations of loxoprofen and its metabolites were not measured, it remains unclear whether intravenous fluid therapy actually enhanced the urinary excretion of these compounds. Furthermore, loxoprofen is also metabolised by CYP3A4/3A5 to an inactive hydroxylated metabolite that competes with trans-OH formation [1]. Therefore, interindividual variability in CYP activity may influence metabolic balance and susceptibility to toxicity.

Drug tolerance to loxoprofen has generally not been reported. Pharmacodynamic drug tolerance to receptor agonists such as opioids typically develops due to opioid adaptive changes in receptor signaling pathways [9]. Because loxoprofen exert their analgesic effects primarily through cyclooxygenase inhibition and suppression of prostaglandin synthesis rather than receptor activation [10], the development of classical pharmacodynamic tolerance is considered unlikely. Pharmacokinetic drug tolerance, which results from altered drug metabolism or elimination [9], has also not been reported for loxoprofen. Therefore, prior exposure to loxoprofen was unlikely to have substantially influenced the pharmacokinetic or toxicological profile observed in this case. Therefore, prior exposure to loxoprofen was unlikely to have substantially influenced the pharmacokinetics or clinical toxicity observed in this case.

In toxic cases, elevated plasma concentrations of loxoprofen and trans-OH persisted, and clinical symptoms developed within 1–2 days after ingestion. In contrast, in the present asymptomatic case, plasma concentrations declined more rapidly, suggesting limited duration of high-level exposure. Therefore, in patients who remain asymptomatic for 3 days after ingestion, hospital discharge may be considered with careful clinical assessment.

## Conclusions

In this case, despite massive ingestion of loxoprofen, no toxicity or organ dysfunction was observed. LC/MS analysis revealed a relatively rapid decline in plasma concentrations of loxoprofen and its metabolites compared with previous reports, suggesting that early clearance may have prevented the development of toxicity.

## Data Availability

The data supporting the findings of this study are available from the corresponding author upon reasonable request.

## Abbreviation

LC/MS: Liquid chromatography–mass spectrometry
